# Augmented Reality-Assisted Craniotomy for Parasagittal and Convexity En Plaque Meningiomas and Custom-Made Cranio-Plasty: A Preliminary Laboratory Report

**DOI:** 10.3390/ijerph18199955

**Published:** 2021-09-22

**Authors:** Nicola Montemurro, Sara Condino, Nadia Cattari, Renzo D’Amato, Vincenzo Ferrari, Fabrizio Cutolo

**Affiliations:** 1 Department of Neurosurgery, Azienda Ospedaliera Universitaria Pisana (AOUP), University of Pisa, 56100 Pisa, Italy; 2 Department of Information Engineering, University of Pisa, 56100 Pisa, Italy; sara.condino@unipi.it (S.C.); renzo.damato@unipi.it (R.D.); vincenzo.ferrari@unipi.it (V.F.); fabrizio.cutolo@endocas.unipi.it (F.C.); 3 EndoCAS Center for Computer-Assisted Surgery, 56100 Pisa, Italy; nadia.cattari@endocas.org; 4 Department of Translational Research, University of Pisa, 56100 Pisa, Italy

**Keywords:** augmented reality, craniotomy, meningioma, surgical planning, laboratory, brain tumor

## Abstract

Background: This report discusses the utility of a wearable augmented reality platform in neurosurgery for parasagittal and convexity en plaque meningiomas with bone flap removal and custom-made cranioplasty. Methods: A real patient with en plaque cranial vault meningioma with diffuse and extensive dural involvement, extracranial extension into the calvarium, and homogeneous contrast enhancement on gadolinium-enhanced T1-weighted MRI, was selected for this case study. A patient-specific manikin was designed starting with the segmentation of the patient’s preoperative MRI images to simulate a craniotomy procedure. Surgical planning was performed according to the segmented anatomy, and customized bone flaps were designed accordingly. During the surgical simulation stage, the VOSTARS head-mounted display was used to accurately display the planned craniotomy trajectory over the manikin skull. The precision of the craniotomy was assessed based on the evaluation of previously prepared custom-made bone flaps. Results: A bone flap with a radius 0.5 mm smaller than the radius of an ideal craniotomy fitted perfectly over the performed craniotomy, demonstrating an error of less than ±1 mm in the task execution. The results of this laboratory-based experiment suggest that the proposed augmented reality platform helps in simulating convexity en plaque meningioma resection and custom-made cranioplasty, as carefully planned in the preoperative phase. Conclusions: Augmented reality head-mounted displays have the potential to be a useful adjunct in tumor surgical resection, cranial vault lesion craniotomy and also skull base surgery, but more study with large series is needed.

## 1. Introduction

In recent years, there has been considerable interest in using augmented reality (AR) head-mounted displays (HMDs), either video see-through (VST) or optical see-through (OST), and mobile devices for AR in the operating room (OR) [1,2,3,4]. Some consumer-level and general purposes OST HMDs, such as the Microsoft^®®^ HoloLens^®®^, can be successfully used for preoperative simulation, for planning and for monitoring patient’s vitals in real-time during surgical procedures. Moreover, even though there are still a few technological and human limitations that prevent high levels of accuracy, an increasing number of research studies propose the use of AR HMDs as a navigation tool for brain biopsy, for brain tumor resection, for anterior and posterior petrosectomy and intracranial aneurysms [5,6,7,8,9,10,11,12,13,14,15].

Among brain tumors, the use of HMDs for surgical resection of intracranial meningiomas, which account for 30% of all primary intracranial tumors [16], has already been proposed [5,13,17]. Considering that their incidence has increased considerably in western societies due to the overall increase in the ageing population and the wide availability of magnetic resonance imaging (MRI) [18], the number of papers reporting intracranial meningiomas treated with AR is expected to grow. In patients with growing and/or symptomatic meningiomas, microsurgical resection is advocated as the main treatment [5]; furthermore, the relationship between the extent of resection (EOR) and the recurrence of meningiomas have been confirmed by many authors [19,20], starting from the pioneering work of Simpson [21]. En plaque meningiomas are a rare type of meningioma characterized by their infiltrative nature, sheet-like growth and invasion of the bone. En plaque meningiomas, which comprise 2–9% of all meningiomas [22], are considered different from their space-occupying counterparts in terms of both their clinical and biologic behaviors, yet their histologic characteristics are similar [23]. For this reason, AR might be a valuable adjunct to surgery when hyperostosis of the sphenoid bone is present, for skull-base meningiomas, and also for cranial vault en plaque meningiomas with hyperostosis as reported in this case.

Treatment of patients with a meningioma with bone infiltration requires removal of the tumor-infiltrated bone and a subsequent cranioplasty. However, cosmetic and neuroprotective reconstructions are necessary after cranioplasty [24]. Cranioplasty after surgical resection of en plaque meningioma, plays a key role in restoring the function and anatomy of the cranial vault. Porous hydroxyapatite (PHA) has received growing attention for its potential in bony integration [25]. Considering that cranioplasty is not exempt from risks, identifying the safest technique is crucial in order to achieve better patient recovery. The use of 3D printing in healthcare has followed a similar path to that of manufacturing, that is, the focus on rapid prototyping has shifted to full-scale production. The rationale behind a preoperatively prepared custom-made bone flap is that having a bone flap ready to be implanted at the time of surgery reduces the surgical time and effort. 

In a recent study, we evaluated the efficacy of an innovative Video and Optical See-Through Augmented Reality Surgical Systems (VOSTARS) HMD [26] in navigating complex craniotomies. The AR navigation platform was quantitatively assessed in terms of accuracy and precision, and the reported results in terms of real-to-virtual target visualization error showed an average accuracy of 1.3 mm (±0.6 mm), which was estimated on landmarks over the skull surface. In addition, the results obtained from the user study of over 30 traced craniotomies, showed that 97% of the lengths of the performed craniotomies were traced within a margin of error of 1.5 mm, and 92% were within a margin of 1 mm. The craniotomy errors were measured by means of manikin-specific 3D-printed templates.

In this study, we present the first laboratory-based results of applying the VOSTARS headset to perform a craniotomy on a custom-made 3D-printed manikin’s head with en plaque cranial vault meningioma and to replace the bone flap obtained during craniotomy with a preoperatively prepared custom-made bone flap.

The aim of this preliminary study is therefore to demonstrate the reliability and feasibility of the VOSTARS surgical navigation platform as a tool to support precise single-stage bone resection and cranioplasty reconstruction for en plaque cranial vault meningioma and for all those lesions affecting the bone that need to be removed and replaced with a customized bone flap of equal size previously prepared.

## 2. Materials and Methods

### 2.1. Case Description

**Figure 1:**
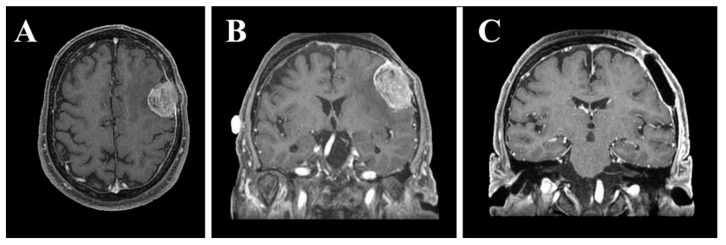
Preoperative axial (**A**) and coronal (**B**) gadolinium-enhanced T1-weighted MRI showing en plaque convexity meningioma. Postoperative coronal gadolinium-enhanced T1-weighted MRI (**C**) showing surgical resection of the tumor and cranioplasty with polymethylmethacrylate.

In this laboratory-based study, we retrospectively used the preoperative and post-operative magnetic resonance imaging (MRI) sequences (Figure 1) of a 68-year-old right-handed male with en plaque meningothelial meningioma (Grade I according to WHO classification) [27], with diffuse and extensive dural involvement, extracranial extension into the calvarium and homogeneous contrast enhancement on gadolinium-enhanced T1-weighted MRI, who underwent surgery at our University Hospital. Meningothelial cells invaded and expanded the left fronto-temporal bones promoting local bone thickening, and in addition, extensive hyperostosis was associated with infiltration of the subarachnoid space, configuring itself as en plaque meningothelial meningioma. For this reason, after tumor resection, the bone flap obtained during craniotomy was not placed and cranioplasty with polymethylmethacrylate (PMMA) cement was performed (Figure 1C). The following paragraphs briefly describe the materials and methods for simulating the craniotomy guided by the VOSTARS system, in a manikin’s head that replicated the anatomy of the above-mentioned surgical case.

### 2.2. VOSTARS HMD-Based Surgical Navigation Platform and Template-Based Registration

A new-concept AR headset was recently developed within the European VOSTARS project (Video and Optical See-Through Augmented Reality Surgical Systems, Project ID: 731974). The overarching goal of the project was to design and develop a new-concept wearable AR platform capable of deploying both video and optical see-through-based augmentation and to validate it as a tool for surgical guidance. The AR platform, which comprises a software framework in combination with an early version of the custom-made headset, is thoroughly described in [28]. This aim of the work was to show the results of an experimental study assessing the efficacy of the AR platform in guiding a simulated tissue incision. The results were very encouraging and supported the claim that the wearable AR framework represents an effective tool in guiding high-precision manual tasks. In [29], the results of a dedicated user study were also promising; the goal was to assess the efficacy and reliability of the AR platform under VST modality in guiding complex 3D trajectory tracing tasks on 3D-printed replicas of planar structures or more elaborated bony structures. Similarly, and as anticipated, encouraging results were also obtained in a study aimed to assess the efficacy of the VOSTARS platform, both in terms of accuracy and precision in navigating complex craniotomies [26].

The AR headset features a computationally efficient and accurate inside-out marker-based tracking mechanism. The optical tracking algorithm provides the pose of an optical frame with respect to the HMD reference system. In this study, the optical frame, comprising three spherical markers, was embedded in a patient-specific template for automatic image-to-patient registration. The template, which is designed to start from the preoperative radiological dataset, was provided with a mating surface that fits the patient’s face over the Rhinion (rhi’), Nasion (n’), Sellion (se’), Suprahelixa interna (sci’), Mid-infraorbital (mio’) in a unique and stable position, thus providing pose registration.

**Figure 2:**
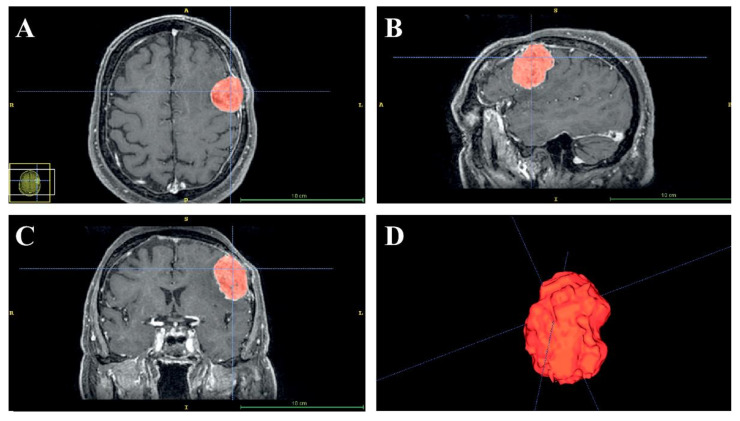
Semi-automatic segmentation of the preoperative axial (**A**), sagittal (**B**) and coronal (**C**) images using the EndoCAS Segmentation Pipeline. A 3D image reconstruction of tumors (**D**).

The preoperative (Figure 2) and the post-operative MRI sequences (axial spoiled gradient recalled acquisition in the steady-state (SPGR) sequences with a 0.5 × 0.5 × 0.6 mm resolution) were segmented using a semi-automatic tool, the “EndoCAS Segmentation Pipeline” (Pisa, Italy) integrated in the ITK-SNAP 1.5 open-source software (University of Pennsylvania, PA, USA), to generate the 3D virtual meshes of the patient face, skull, brain and meningioma. 

**Figure 3:**
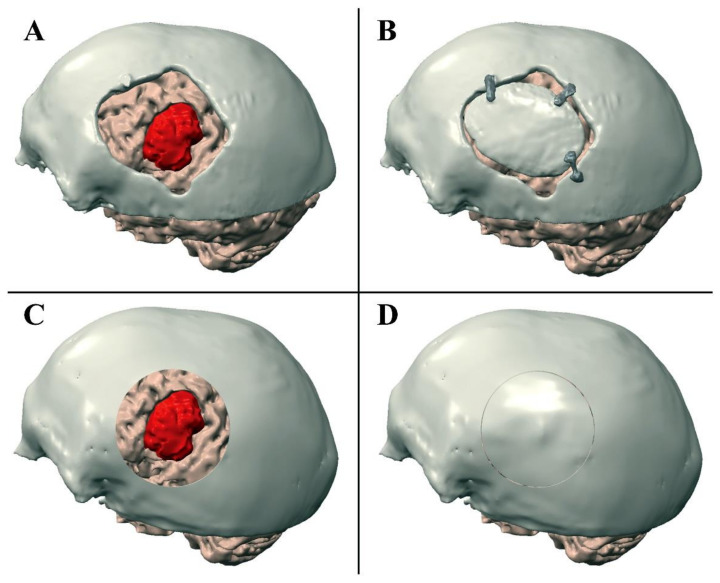
Performed craniotomy (**A**,**B**) and VOSTARS planning (**C**,**D**). (**A**) Shows the post-operative skull model was registered to the preoperative brain model. (**B**) Shows the position of the bone flap cranioplasty with polymethylmethacrylate. (**C**) Shows the craniotomy planned with VOSTARS. (**D**) Shows the result of the ideal post-operative bone flap cranioplasty on the manikin’s head.

**Figure 4:**
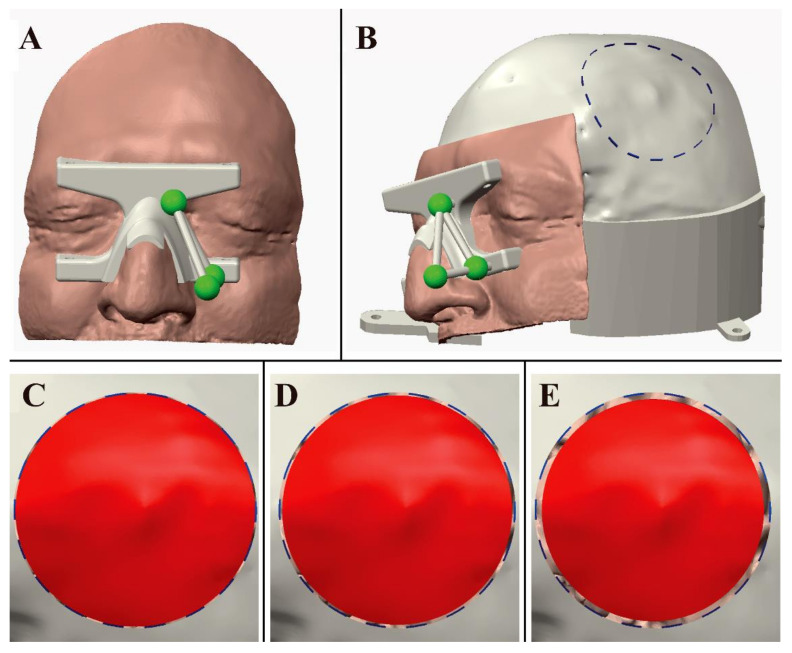
(**A**) Design of the patient-specific registration template and of the manikin’s head. (**B**) Design of the manikin’s head with planned circular craniotomy trajectory. (**C**–**E**) Shows three bone flaps with a gap that decreases 0.5 mm (**C**), 1 mm (**D**), 2 mm (**E**) from the ideal trajectory.

Preoperative and post-operative meshes were manually registered to visualize the position of the meningioma with respect to the performed craniotomy (Figure 3). Preoperative meshes were refined via the open-source software MeshLab by performing a few optimization stages (e.g., removal of non-manifold edges and vertices, holes filling, and finally, mesh filtering). Creo^®®^ Parametric CAD software (PTC Creo, Las Vegas, NV, USA) was used to design a patient-specific manikin, replicating a portion of the patient’s skull and face (Figure 4A,B), to plan the ideal craniotomy (Figure 3C,D), and design the bone flaps (Figure 4C–E) as well as the patient-specific registration template (Figure 4A). The patient-specific head manikin, the template, and the bone flaps were printed with a 3D rapid prototyping machine, the Stratasys Dimension Elite, a professional Fused Deposition Modeling (FDM) 3D printer made by Stratasys (minimum layer thickness, 0.127 mm).

The planned circular craniotomy trajectory (Figure 4B) was represented by a dashed curve (0.5 mm thickness) and exported as a Virtual Reality Modeling Language (VRML) file model to be loaded by the software framework and displayed by the AR headset. As for the bone flap, different sizes were designed to evaluate the precision of a craniotomy obtainable by exploiting the VOSTARS navigation platform. More specifically, five bone flaps were designed with a radius decreasing in steps of 0.5 mm from 60 cm (0.5 mm smaller than the radius of the ideal craniotomy) to 57.5 cm (2.5 mm smaller than the radius of the ideal craniotomy) (Figure 4C–E).

### 2.3. AR Visualization Modalities

**Figure 5:**
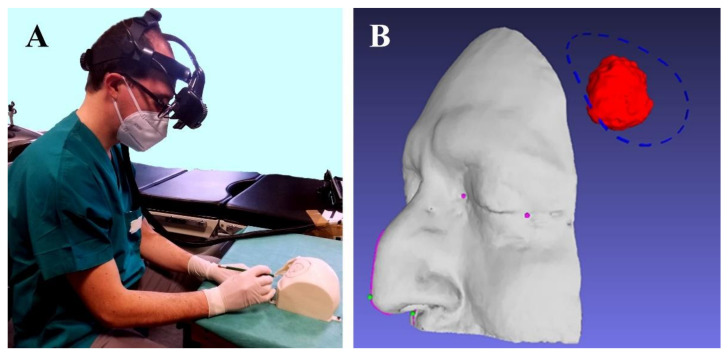
(**A**) The neurosurgeon drawing the craniotomy line on the manikin’s head with a pencil. (**B**) Shows the virtual content to be displayed by VOSTARS: craniotomy, meningioma, medial and lateral canthi, pronasal point at the anterior nasal apex and nasospinal and profile of the patient’s nose.

**Figure 6:**
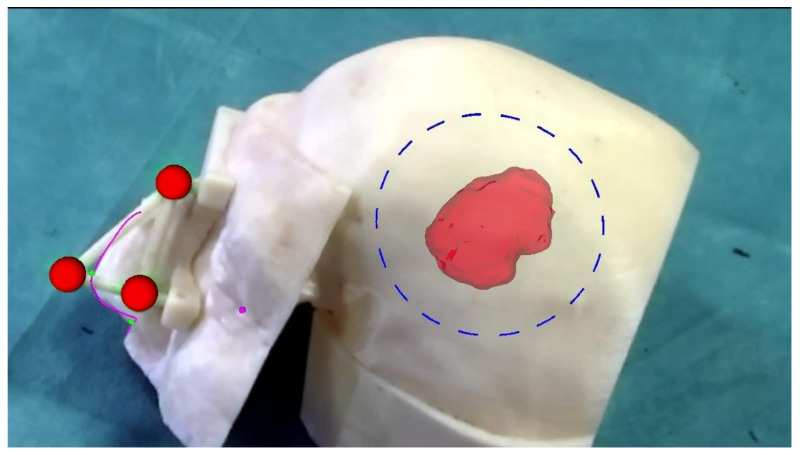
Augmented reality scene with 3D reconstruction of the meningioma (the semi-transparent red structure), the planned craniotomy (the blue dashed line), the optical markers (the red spheres), the reference landmarks (the small pink and green spheres) and the nose profile (the pink line). The last two virtual elements are used as reference landmarks to verify the accuracy of the image-to-patient registration. The ideal pre-operative and intra-operative procedural steps are outlined in Figure 7.

The virtual content that was used to perform the craniotomy was a virtual craniotomy trajectory whose size, shape, and location were dictated preoperatively through the CAD software. The AR application also showed a 3D reconstruction of the meningioma, the virtual homologous of the optical markers, few virtual spheres located at anatomic landmarks (lateral canthi, pronasal point at the anterior nasal apex and nasospinal) and the profile of the patient’s nose. (Figure 5) The virtual spheres as well as the profile of the patient’s nose were used to verify the correct placement of the registration template by directly assessing the accuracy of the associated AR overlay 4 (Figure 6).

**Figure 7:**
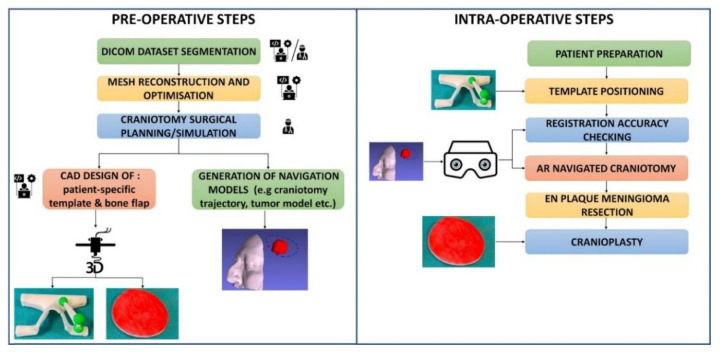
Description of the main pre- and intra-operative procedural steps of the operative workflow to set up the AR-based neurosurgical navigation platform.

## 3. Results

**Figure 8:**
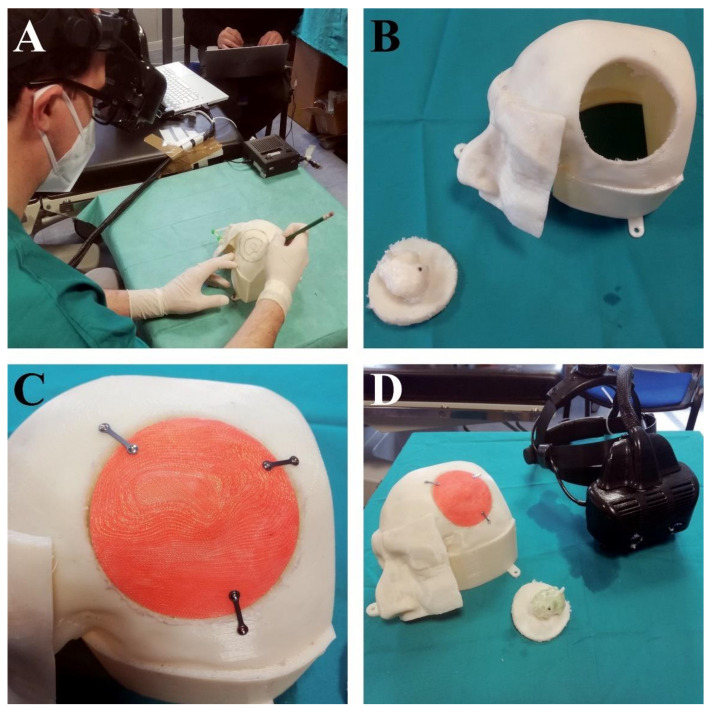
(**A**) The neurosurgeon drew the craniotomy line on the manikin’s head with a pencil by following the augmented reality craniotomy line. (**B**) Shows the craniotomy that was performed. (**C**) Shows there was no gap between the previously prepared custom-made bone flap and the manikin’s head, demonstrating an error of less than ±1 mm in task execution. (**D**) The VOSTARS HMD and the manikin’s head with the new bone flap.

By using the VOSTARS HMD system fitted on the surgeon’s head, a short setup (less than one minute) was needed to check the tracking robustness and the accuracy of the associated AR overlay. The neurosurgeon reported good wearability and ergonomics for the headset. Wearing the VOSTARS headset and by following the displayed craniotomy line, the neurosurgeon was able to draw the craniotomy line on the left side of the head manikin with a pencil (Figure 8A). Then, using a piezoelectric saw (Piezosurgery 3 by Mectron spa, Carasco, Italy), the surgeon performed the craniotomy on the head of the manikin, and removed the bone flap with the extra-axial intradiploic lesion (which mimicked an en plaque meningioma) (Figure 8B). At this point, the five previously prepared custom-made bone flaps were placed on the head of the manikin to select the best fitting one. The bone flap with the narrowest margin with respect to the planned craniotomy (Figure 8C) (i.e., with a radius 0.5 mm smaller than the radius of ideal craniotomy) fitted very well, thus demonstrating that an error lower than ±1 mm was achieved in the task execution.

## 4. Discussion

In the last few years, the guidance accuracy and precision of AR has been refined, which allows healthy and diseased tissue to be distinguished; this improves the intuitiveness of the guidance visualization, and the surgical outcomes [4]. AR, or the fusion of virtual information with the real environment, allows subsurface anatomy to be superimposed on to a surgeon’s live endoscopic or microscopic view by using concurrent navigation, which facilitates neurosurgical guidance [30,31]. The first use, although rudimentary, of AR for neurosurgical guidance dates back to the late 1980s [32]. However, the use of virtual and AR technology in neurosurgery started to catch on less than 20 years ago with the overarching goals being to help surgeons to achieve optimal tumor resection, to reduce the size of the craniotomy, to improve the clinical outcomes of patients, as well as to act as a new tool for neurosurgical education [23,33,34,35,36,37]. In addition, the correct identification of the brain’s functional areas during surgery helps surgeons to reduce the damage to normal brain tissue [1,5,6].

Neuronavigation systems that make use of AR have been the focus of much research in the last couple of decades. The adoption of HMD technology in the surgical room has attracted a lot of research interest lately, and studies on AR HMDs provide insight into the disruptive potential of AR in the surgical field as it allows ergonomic, intuitive, and three-dimensional realization of preoperative and intraoperative information.

In various cadaveric and live surgical series, the accuracy and precision of AR are directly related to that of the surgical navigation system. Adequate accuracy has been reported in identifying the facial nerve during robot-assisted cochlear implantation [38], functional endoscopic sinus surgery [39], inter-cavernous carotid dissection during anterior skull base surgery and brain tumor localization in cranial procedures [40,41]. AR has been hypothesized to facilitate faster mental three-dimensional model building; the surgeon no longer has to extrapolate two-dimensional information from imaging into his/her own three-dimensional construct [42]. This is especially important in a relatively small, anatomically dense area such as the lateral skull base and cerebellopontine angle. A better mental three-dimensional model is likely to lead to improved situational awareness and a lower likelihood of critical errors [1,43]. In addition to facilitating the approach to a complex craniotomy and skull base lesions, AR can facilitate preoperative planning and support the surgeon in performing a precise and accurate craniotomy that fits perfectly with the custom-made bone flap. In a recent work [44], the results of a cadaveric study confirm the results obtained by our pilot study. The work aimed to assess the efficacy of a novel image-guided neuronavigation software in performing single-stage free-hand craniotomies and cranioplasty reconstructions. The reported surgical precision measured in the virtual and performed craniotomies was similar to that obtained in our preliminary test (1.1 mm mean precision), whereas the reported surgical accuracy, which was affected by a registration error, was substantially higher (mean of 4.5 mm) than those obtained by our in vitro study [26]. According to the results of our previous study, the accuracy provided by the VOSTARS system and measured through 3D printed templates was ≤1.5 mm for 97% of the traced craniotomies lengths).

Modeling and simulation of soft tissue deformation is a fundamental research topic in the area of neurosurgical simulation. Surgical simulation requires realistic and real-time modeling. Miller et al. [45] proposed modeling approaches related to geometry creation, boundary conditions, loading and material properties, and advocated the use of fully nonlinear modeling approaches capable of capturing very large deformations and the nonlinear material behavior of the brain for neurosurgery. Similarly, Zhang et al. [46] recently published a review of real-time deformable models for surgical simulation. These models were classified into three categories: the heuristic modeling methodology, continuum-mechanical methodology and other methodologies. Zhang [47] reported a novel algorithm for total Lagrangian explicit dynamics (TLED) FE using a direct Jacobian formulation applied to brain-shift non-rigid registration in craniotomy. We are working on using the same platform for surgery, in this case it will be necessary to incorporate the biomechanics of the brain/skull base and real-time surgical simulation for AR.

Fick et al. [48] included thirty-five studies in their review and reported five methods for assessing accuracy. They concluded that AR technology seems capable of achieving an accuracy comparable to conventional infrared neuronavigation systems. In this study, we presented the first laboratory-based results of the application of the innovative VOSTARS headset, designed to guide complex craniotomies and tumor resection in neurosurgical navigation, to draw a precise craniotomy in order to perfectly fit a previously prepared bone flap. Overall, the VOSTARS AR headset showed enormous potential to help the surgeon in identifying tumor locations, delineating the planned craniotomy, reducing the risk of injury to invisible structures and helping the surgeon in a cranioplasty with a previously prepared custom-made bone flap. Moreover, AR visualization can help the surgeon to understand the relative proximities between tumor, eloquent areas and surrounding brain parenchyma.

Compared to other HMDs, such as smart glasses for neurosurgical navigation as reported by Maruyama et al. [1], which are limited because the overlaid images are only seen by the surgeon wearing the glasses, in the VOSTARS system the AR images can also be observed by other surgeons and surgical nurses on an external display located in the operating room, as well as remotely. Compared to mobile devices for AR, which have many advantages including the fact that they are easy to move around and can be draped in sterile bags, which allows for continuous use throughout a procedure using a touch screen [4], VOSTARS system have proved to be effective in guiding skin incision, craniotomy and lesion targeting, and are also useful to facilitate training in brain tumor resection procedures.

In our experiment, the geometric matching between the physical model of the bone flap and the performed craniotomy depends on the quality of the preoperative imaging (e.g., the resolution of the MRI dataset), on the accuracy and precision of the segmentation (i.e., the dimensional correspondence of the segmented 3D model of the skull and printed skull of the patient), on the accuracy of the 3D printer in reproducing the physical models of bone flap, the patient-specific manikin, and the patient-specific template, on the accuracy of the patient-specific template design and positioning and of the VOSTARS HMD-based surgical navigation platform, and finally, on the ability of the user in performing an accurate craniotomy following the AR information. Similarly, in a real surgical scenario, the overall accuracy and precision of the obtained results will depend on the accuracy and precision of the segmentation, on the accuracy of 3D printing, which will only affect the fabrication of the bone flap here, and the specific patient template. In addition, the overall accuracy will also depend on the accuracy of the patient-specific template design and positioning, on the accuracy and precision of the VOSTARS HMD-based surgical navigation platform and on the ability of the user in performing the craniotomy following the AR information.

These preliminary results support a structured study to prove actual craniotomy accuracy and clinical effectiveness on cadavers and/or on real patients. The use of virtual reality-based simulation systems has been highlighted in a previous article as a concept for future development in the neurosurgical operative environment [3,48]. This underscores its acceptance and importance as a neurosurgical operative aid and its potential to improve safety in neurosurgical procedures in the future [41,49]. The use of 3D stereoscopic AR visualization in the preoperative as well as in the intra-operative setting is deemed to improve the surgeon’s understanding of the anatomy and facilitate the tasks involved [41,50,51,52,53]. 

This system has some limitations. The marker-based tracking modality and the automatic image-to-patient registration based on the patient-specific template can potentially suffer from the effects of brain shift resulting in reduced AR visualization accuracy if used to support neurosurgical procedures inside the brain, as with other neuronavigation systems involving non-rigid structures [49,54,55]. As an alternative, future studies need to develop automated and intra-operative image reconstructions obtained from 3D scans of the anatomy to be combined with preoperative and intra-operative radiological images.

## 5. Conclusions

The results of this study show the potential of a recently developed AR headset in neurosurgery through a laboratory evaluation of the achievable efficacy in visualizing targets on the skull and aiding surgeons in performing craniotomies and in repositioning a custom-made bone flap. The presented AR neurosurgical navigation platform provides an unprecedented 3D visualization of both the surgical field and virtual elements, improves the depth-perception of the augmented scene, and proved to be capable of effectively supporting the surgeon in performing highly precise and accurate craniotomies. The results obtained in terms of craniotomy precision are promising and encourage us to take the next step, such as testing in the operating room on cadavers, and finally, on real patients. We expect this system to be well received in neurosurgical practice. The ergonomic aspects were a high priority during the development of the headset. We also showed the efficacy and reliability of a simple and accurate system for automatic image-to-patient registration. In the future, we will compare the performance of this method with other traditional registration modalities that do not require the fabrication of a patient-specific template. A more structured user study is needed to record the specific ways in which AR plays a role in each procedure and how it may change decision making. 

## Data Availability

Data sharing not applicable.
